# Correction to ‘‘Heat Loss Quantification and Heat Transfer in Rotary Kilns for Calcination and Clinker Formation: From Combustion and Electrification at 150 kW to Industrial Scale’’

**DOI:** 10.1021/acs.iecr.5c04150

**Published:** 2025-10-15

**Authors:** Ibrahim Qasim, Adrian Gunnarsson, Fredrik Normann, Bodil Wilhelmsson, Alexander Zether, Klas Andersson

In Figure 8a, the numerical values for sensible heat and heat of reaction were inadvertently interchanged. The sensible heat value was reported as 14 and should be corrected to 46, while the heat of reaction was reported as 46 and should be corrected to 14. Similarly, in Figure 8b, the sensible heat value was reported as 8 and should be corrected to 37, while the heat of reaction was reported as 37 and should be corrected to 8. The value for bed material energy, which is the central quantity for analysis and discussion, was correctly reported. The data sets, calculations, and results presented in the manuscript remain unchanged, and the overall analysis and conclusions are unaffected.

**Figure 8:**
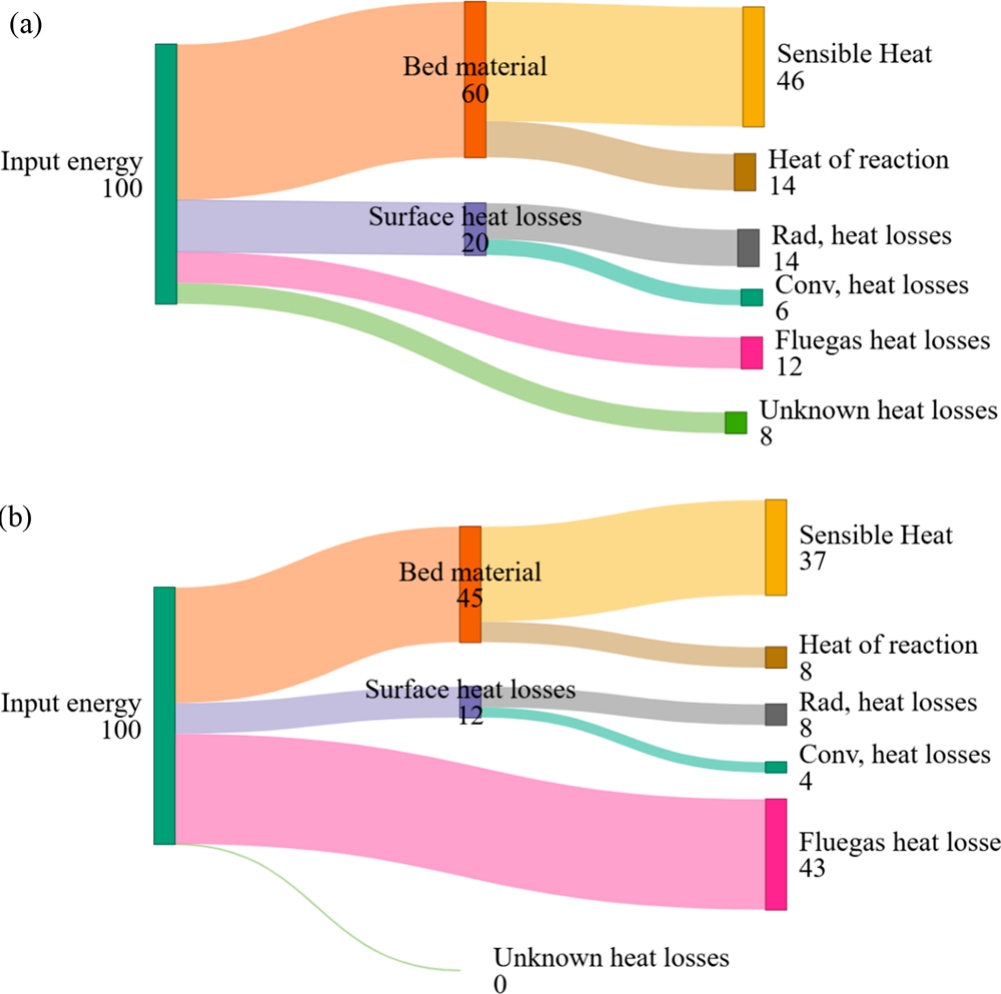


This correction is issued solely to prevent any possible confusion regarding the labeling of Figure 8.

